# Supplementary material for: Iterative improvement in the automatic modular design of robot swarms
Source: PeerJ Comput Sci. 2020 Dec 7;6:e322. doi: 10.7717/peerj-cs.322 (PMC7924708; doi:10.7717/peerj-cs.322)
Supplement: Supplemental Information 3 [file peerj-cs-06-322-s003.zip › argos3/doc/api/standalone/a00401.html]

ARGoS: core/utility/plugins/vtable.h File Reference


- Main Page
- Related Pages
- Namespaces
- Classes
- Files

- File List
- File Members

# core/utility/plugins/vtable.h File Reference

`#include <vector>`  
`#include <cstddef>`  
`#include <argos3/core/utility/logging/argos_log.h>`  

Include dependency graph for vtable.h:

Go to the source code of this file.

|  |  |
| --- | --- |
| Classes | |
| struct | argos::STagCounter< BASE > |
|  | Holds the value of the last used tag. More... |
| struct | argos::STagHolder< DERIVED, BASE > |
|  | Holds the value of the tag associated to `DERIVED`. More... |
| struct | argos::EnableVTableFor< BASE > |
|  | Helper to make a class hierarchy vtable-enabled. More... |
| class | argos::CVTable< CONTEXT, BASE, FUNCTION > |
|  | The actual vtable. More... |
| Namespaces | |
| namespace | argos |

|  |  |
| --- | --- |
|  | The namespace containing all the ARGoS related code. |

| Defines | |
| #define | ENABLE\_VTABLE() |
|  | This macro enables the vtable for a specific class. |
| #define | INIT\_VTABLE\_FOR(BASE) |
| Functions | |
| template<typename DERIVED , typename BASE > | |
| size\_t | argos::GetTag () |
|  | Returns the value of the tag associated to `DERIVED`. |
| template<typename CONTEXT , typename BASE , typename FUNCTION > | |
| CVTable< CONTEXT, BASE,   FUNCTION > & | argos::GetVTable () |
|  | Function that returns a reference to the static vtable. |

---

## Define Documentation

|  |  |  |  |  |
| --- | --- | --- | --- | --- |
| #define ENABLE\_VTABLE | ( |  | ) |  |

**Value:**

```
virtual size_t GetTag() {                    \
      return GetTagHelper(this);                \
   }
```

This macro enables the vtable for a specific class.

Definition at line 146 of file vtable.h.

|  |  |  |  |  |  |
| --- | --- | --- | --- | --- | --- |
| #define INIT\_VTABLE\_FOR | ( | BASE |  | ) |  |

**Value:**

```
struct SVTableInitializerFor ## BASE {       \
      SVTableInitializerFor ## BASE() {         \
         GetTag<BASE, BASE>();                  \
      }                                         \
   } sVTableInitializerFor ## BASE;
```

Definition at line 204 of file vtable.h.

---

Generated on 10 Jul 2018 for ARGoS by 
 1.6.1
